# Olmesartan Improves Hepatic Sinusoidal Remodeling in Mice with Carbon Tetrachloride-Induced Liver Fibrosis

**DOI:** 10.1155/2022/4710993

**Published:** 2022-08-26

**Authors:** Ying Wu, Xue Ge, Si-Ning Wang, Chun-Qing Zhang

**Affiliations:** ^1^Department of Gastroenterology, The Second Hospital, Cheeloo College of Medicine, Shandong University, Jinan, Shandong, China; ^2^Department of Intensive Care Unit, The Second Hospital, Cheeloo College of Medicine, Shandong University, Jinan, Shandong, China; ^3^Department of Gastroenterology, Shandong Provincial Hospital, Cheeloo College of Medicine, Shandong University, Jinan, Shandong, China

## Abstract

**Aim:**

In mice with liver fibrosis produced by carbon tetrachloride (CCl_4_), the effects of olmesartan on intrahepatic angiogenesis and sinusoidal remodeling will be evaluated.

**Methods:**

By injecting CCl_4_ into the peritoneal cavity, we established a mouse model of liver fibrosis. Using Sirius red and Masson trichrome staining, the extent of liver fibrosis in the animals was determined. Using immunohistochemical labeling and western blotting, the level of *α*-smooth muscle actin (*α*-SMA) expression, a characteristic of hepatic stellate cell activation, was assessed. Electron microscopy was used to determine the effect of olmesartan on hepatic sinusoidal capillarization, and immunohistochemical labeling was used to determine the expression levels of endothelial and basement membrane proteins in mouse liver tissues. Platelet-derived growth factor (PDGF), IL-10, vascular endothelial growth factor (VEGF), and angiotensin II levels in mouse serum were measured by Luminex multifactor analysis and ELISA. Olmesartan's effect on the angiotensin II type 1 receptor (AT1R) and the VEGF receptor (VEGFR) was evaluated using western blotting.

**Results:**

Olmesartan reduced CCl_4_-induced inflammatory cell infiltration and collagen deposition to alleviate liver fibrosis. *α*-SMA expression was decreased, and HSC activation was inhibited in mouse liver tissues by olmesartan treatment. In addition, hepatic sinusoidal capillarization was improved under the action of olmesartan. The expression of collagen IV, fibronectin, CD31, and von Willebrand factor (VWF) in the olmesartan group was also markedly downregulated. In fibrotic mice, olmesartan medication decreased the levels of PDGF, VEGF, and angiotensin II, but it increased the level of IL-10. Moreover, olmesartan reduced the expression of VEGFR-1, VEGFR-2, and AT1R relative to CCl_4_-induced liver fibrosis.

**Conclusions:**

In mice with CCl_4_-induced fibrosis, olmesartan lowers angiogenesis and improves hepatic sinusoidal remodeling, according to our findings. By acting on the angiotensin II-AT1R-VEGF axis, this is achieved.

## 1. Introduction

Portal hypertension can lead to splenomegaly, ascites, and esophageal varices and is the prime cause of high mortality in cirrhosis patients [1]. In liver fibrosis and cirrhosis, excessive extracellular matrix (ECM) deposition, the formation of regenerative nodules, and construction and remodeling of hepatic sinusoidal vessels lead to increased sinus resistance and portal pressure [2]. Sinusoidal resistance, fibrosis, and portal hypertension are all linked to intrahepatic angiogenesis and sinusoidal remodeling, according to study [1]. The emergence of neoangiogenesis and abnormal vascular structures in the liver are closely connected with the progression of chronic liver disease [3, 4]. Understanding the relationship between angiogenesis and fibrogenesis may give a novel treatment strategy for liver fibrosis and portal hypertension [5, 6].

The complex vascular structure known as the hepatic sinusoid is comprised of hepatic stellate cells (HSCs) whose cell processes surround the sinusoid, liver sinusoidal endothelial cells (LSECs) that constitute the sinus wall, Disse space between LSECs and hepatocytes, and Kupffer cells. HSCs and LSECs play key roles in increasing liver vascular resistance. Activated HSCs contribute to fibrogenesis and the reconstitution of intrahepatic structures through cell proliferation and ECM production [1, 7, 8]. In addition, LSEC phenotypic modifications and increased number of contractile HSCs surrounding LSECs are hallmarks of sinusoidal vascular remodeling [9]. In liver fibrosis and cirrhosis, LSECs lose their fenestrae, totally form a continuous basement membrane, and undergo capillarization [10]. The formation of LSEC capillarization accelerates HSC activation and fibrogenesis, and the disappearance of the normal filtration barrier results in changes in liver microcirculatory functions [4]. On the other hand, activated HSCs secrete angiogenic molecules that stimulate LSECs, thereby promoting angiogenesis [1, 11]. The amelioration of liver fibrosis by particular angiogenesis-inhibiting drugs, such as those that target vascular endothelial growth factor receptor-2 (VEGFR-2), demonstrates the importance of angiogenesis in fibrogenesis [1, 12].

The renin-angiotensin system (RAS) is vital for maintaining electrolyte balance and blood pressure stability [13, 14]. The angiotensin II type 1 receptor (AT1R) is one of the RAS components that activated HSCs may express [15]. When liver fibrosis occurs, local intrahepatic angiotensin II levels increase and angiotensin II binds with AT1R on the surface of HSCs to promote HSC activation, proliferation, and contraction through multiple signal transduction pathways [16, 17]. Angiotensin II receptor blockers have been shown in animal experiments to modulate the RAS in the treatment of liver fibrosis; however, the specific molecular mechanism is still unclear [18, 19]. Angiotensin II is a potent vascular smooth muscle cell growth factor that, according to studies [20, 21], upregulates VEGF and induces angiogenesis. In addition, the AT1R antagonist candesartan has been shown to diminish angiogenesis in hepatocellular carcinoma by blocking the VEGF pathway [20]. The present study examined the effect of the AT1R antagonist olmesartan on the inhibition of angiogenesis and improvement of sinusoidal remodeling via the RAS in fibrotic mice.

## 2. Materials and Procedures

### 2.1. Animal Model Construction

MedChemExpress (NJ, USA) provided olmesartan for our experiment. Shandong University's Experimental Animal Center (Jinan, China) supplied 30 adult male C57BL/6 mice, which were housed under constant environmental temperature (23–25°C). All the animals were provided with a consistent diet and unrestricted water. The animal care and utilization committee at Shandong University approved the experimental protocols.

For this study, a mouse model of liver fibrosis was developed using our previous methods [22]. Three types of mice were randomly selected from the animal population: (1) a normal control category, in which mice received intraperitoneal injections of olive oil (5 ml/kg) twice weekly for 8 weeks, (2) the carbon tetrachloride (CCl_4_) category, in which fibrosis was induced by intraperitoneal injections of CCl_4_ in olive oil (30 percent, 5 ml/kg) twice weekly for 8 weeks, and (3) the CCl_4_ + olmesartan (5 mg/kg/d) category, in which mice were administered CCl_4_ (30 percent, 5 ml/kg) for the first 2 weeks and then administered both drugs for the next 6 weeks. The survival percentage for mice at the completion of the experiment was 80%.

### 2.2. Cytokine ELISA

The blood samples were obtained after the mice had been sedated and centrifuged for 10 minutes at 3000 rpm, and the supernatants were collected. In accordance with the manufacturer's instructions, an ELISA kit (RayBiotech, GA, US) was used to quantify the quantity of angiotensin II in each category's serum. Using a scanning MultiWell spectrophotometer, the absorbance at 450 nm was measured (BioRad Model 550, CA, US). ELISACalc software was used to plot the standard curve and calculate the angiotensin II concentrations in samples. The results were analyzed with GraphPad Prism 7.0.

### 2.3. Luminex Analysis

Blood samples from mice were centrifuged and collected using the method described above. The relevant biomarkers (platelet-derived growth factor (PDGF), IL-10, and VEGF) in mouse serum were measured following the instructions using a R&D LXSAMSM-09 Luminex System. The data were analyzed with GraphPad Prism 7.0.

### 2.4. Histopathological Examination

Paraffin slices of liver tissue were stained with masson trichrome, sirius red, and hematoxylin and eosin using conventional procedures. The METAVIR scale rated the severity of liver fibrosis (0 for no fibrosis, 1 for portal fibrosis, 2 for periportal fibrosis, 3 for bridging fibrosis, and 4 for cirrhosis) [23].

### 2.5. Immunohistochemistry

The liver slices were deparaffinized, serially dehydrated with ethanol, and subjected to the heat-induced antigen retrieval process. The sections were then blocked, followed by overnight primary antibody incubation at 4°C. *α*-Smooth muscle actin (*α*-SMA) (1 : 400, Abcam, MA, US), collagen IV (1 : 1000, Abcam, MA, US), fibronectin (1 : 2000, Abcam, MA, US), CD31 (1 : 2000, Abcam, MA, US), and VWF (1 : 200, Proteintech, IL, US) were the primary antibodies used in this study. The sections were stained with diaminobenzidine and hematoxylin after being subjected to the biotinylated secondary antibody. Under a light microscope, the positively stained areas appeared brownish-yellow. The results were analyzed with Image-Pro Plus 6.0.

### 2.6. Transmission and Scanning Electron Microscopy (TEM and SEM)

Fresh mouse liver tissue slices were fixed in special fixation solution for two hours and then postfixed in 1 percent osmic acid buffer solution for two hours. After ethanol dehydration, the samples were infiltrated, embedded, and aggregated. Following this, the resin blocks were chopped into thin sections and coloured with an alcohol solution containing 2% uranium acetate. TEM (HT7800/HT7700, Hitachi, Tokyo, Japan) was used to inspect and photograph the materials.

For scanning electron microscopy, fixed tissues were serially dehydrated in ethanol and dried in a Quorum K850 critical point drier. Following conductive treatment in an IXRF MSP-2S ion sputtering analyzer, the images were captured with a Hitachi SU8010 scanning electron microscope.

### 2.7. Western Blot Analysis

To conduct a western blot study on liver tissue proteins, the protein concentration of each sample was determined using a BCA protein quantification kit. The protein samples were electrophoresed on 8–12 percent SDS-PAGE gels prior to being transferred to polyvinylidene fluoride filter membranes. The membranes with protein were first treated for the duration of a night at 4°C with primary antibodies before being blocked for one hour. After incubation with secondary antibodies and washing with TBST buffer, the protein bands were examined using a Millipore-enhanced chemiluminescence test kit. Using ImageJ, the optical densities of the bands were analyzed.

### 2.8. Statistical Analysis

Mean and standard deviation are used to illustrate the data. For the statistical analysis, the one-way ANOVA or Student's *t*-test was used. For all tests, values of *P* < 0.05 were considered statistically significant.

## 3. Results

### 3.1. Effects of Olmesartan on Hepatic Fibrogenesis and Injury

**Figure 1:**
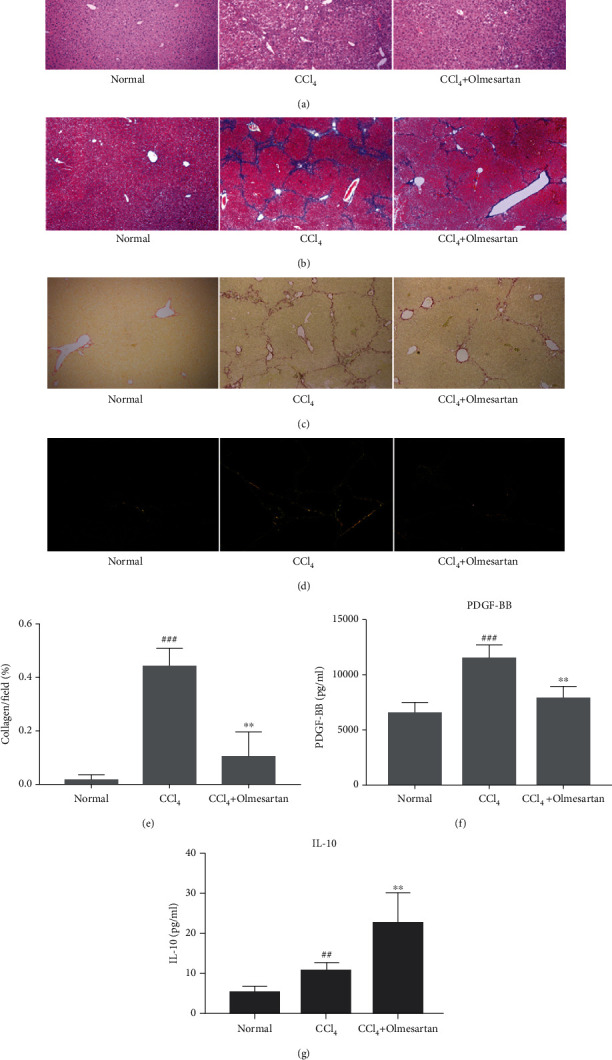
Effects of olmesartan on hepatic fibrosis and collagen production in mice treated with CCl_4_. (a, b) H&E and Masson trichrome dyeing of liver tissues in different groups of mice were photographed. (c–e) The sirius red staining (polarized light shown in (d)) of liver tissues in different groups was observed, and the quantity of collagen production in each group was determined. Magnification ×100. (f, g) Serum PDGF-BB and IL-10 levels were measured. ^###^*P* < 0.001 versus normal; ^##^*P* < 0.01 versus normal; ^∗∗^*P* < 0.01 versus CCl_4_.

Olmesartan's effect on CCl_4_-induced hepatic fibrosis was determined using pathological staining (Figures 1(a)–1(d)). In the CCl_4_ group, the liver architecture was much more disordered than that in the normal group and hepatic sinusoids could no longer be identified. After 8 weeks of CCl_4_ injections, liver tissues exhibited increased inflammation and fibrous connective tissues (Figure 1(a)) (METAVIR more than 2). Masson trichrome and Sirius red staining revealed visible collagen production and the establishment of fibrous septa across the portal regions in the CCl_4_ group. However, olmesartan alleviated these changes (Figures 1(b) and 1(c)) (METAVIR no more than 2). Under polarized light, Sirius red staining revealed that mice administered just CCl_4_ had much more collagens I and III (shown in red and green, respectively) deposited in their livers. In contrast, olmesartan medication decreased collagen production in the periportal zones, interlobular septum, and perisinusoidal spaces (Figures 1(c)–1(e)).

PDGF signaling is crucial during HSC activation and angiogenesis. The results demonstrated that olmesartan decreased the expression of PDGF-BB, although serum PDGF-BB levels were higher in CCl_4_-injected fibrotic mice than in normal mice (Figure 1(f)). IL-10 is a potential anti-inflammatory factor that can inhibit the expression of a variety of proinflammatory mediators. According to the results, IL-10 expression levels in the olmesartan group were notably higher than those in the CCl_4_ group (Figure 1(g)).

### 3.2. Effects of Olmesartan on HSC Activation

**Figure 2:**
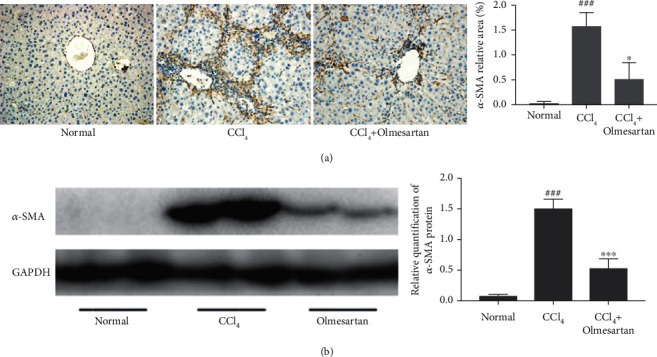
Effects of olmesartan on HSC activation in CCl_4_-induced fibrotic mice. (a) *α*-SMA immunohistochemistry was performed on mouse liver tissues from each group (magnification ×200). (b) The levels of *α*-SMA expression in mouse liver tissues were determined by western blot. ^###^*P* < 0.001 versus normal; ^∗∗∗^*P* < 0.001 versus CCl_4_; ^∗^*P* < 0.05 versus CCl_4_.

After HSC activation, the expression of *α*-SMA, a critical marker associated with liver fibrosis, is increased. According to immunohistochemical labeling, *α*-SMA-positive cells were located along the endothelia of hepatic sinusoids in the CCl_4_-administered mice. The sinusoidal walls of unaffected mice lacked any visible positive staining. Olmesartan therapy produced significantly less *α*-SMA-positive cells than the CCl_4_ group (Figure 2(a)). In addition, western blot test findings revealed that olmesartan medication lowered the protein expression of *α*-SMA (Figure 2(b)).

### 3.3. Effects of Olmesartan on the Hepatic Sinusoidal Capillarization of Mice

**Figure 3:**
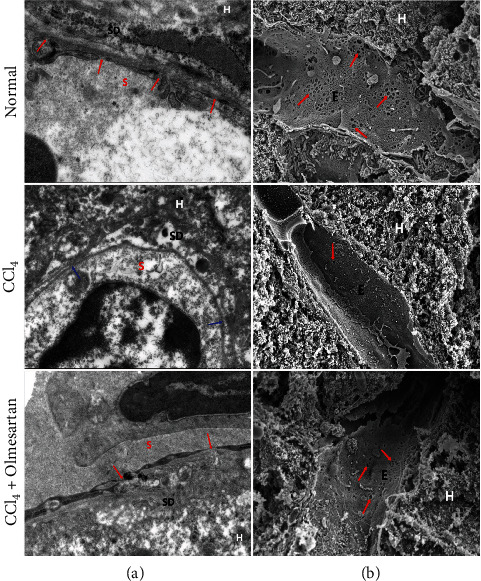
(a) A transmission electron micrograph depicting hepatic sinusoids in mice liver tissues. Representative images showed changes in the endothelial fenestrae and capillarization of the sinusoid in fibrotic mice. The fenestration structure of the mice in the CCl_4_ group disappeared, and a continuous basement membrane was formed (as indicated by blue arrows), while multiple fenestrae were seen in the hepatic sinusoids of the normal mice and the olmesartan-treated mice (as shown by red arrows). S is the sinusoid, SD is the Disse space, and H is the hepatocyte. (b) A scanning electron micrograph of fenestrae in the sinusoidal endothelium of the liver of mice (as indicated by red arrows). There is defenestration of the endothelium in the CCl_4_ group. E is the endothelial cell, and H is the hepatocyte. Magnification ×10 k.

Fenestrae are round or pore-like features that penetrate the cytoplasm of sinusoidal endothelial cells, as described in reference [24]. According to TEM and SEM detections of mouse hepatic sinusoids, the LSECs of the normal control group contained plenty of fenestrae with a discontinuous basement membrane. In mice administered CCl_4_, the fenestrae of the hepatic sinusoids shrank or disappeared and the basal side of LSECs established a continuous basement membrane. However, mice treated with olmesartan showed more fenestrae than those treated with CCl_4_ alone (Figure 3).

**Figure 4:**
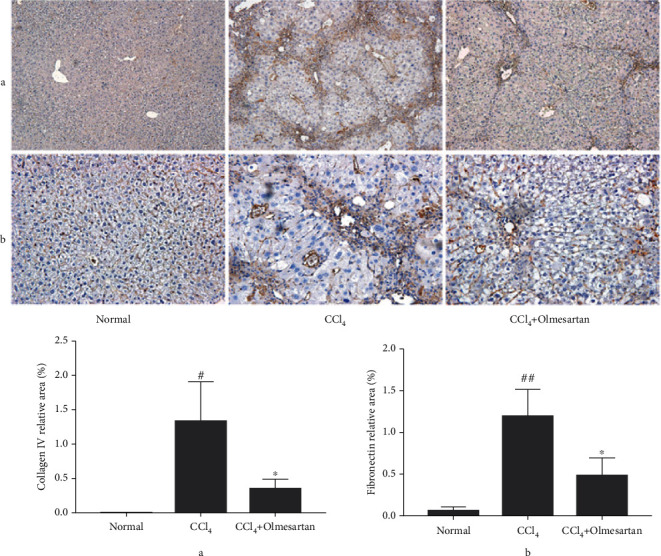
The effects of olmesartan on the proteins that compose the liver's basement membrane in fibrotic mice. (a, b) Immunohistochemistry found collagen IV and fibronectin in the liver tissues of mice from each group (magnification ×200). ^##^*P* < 0.01 vs normal; ^#^*P* < 0.05 versus normal; ^∗^*P* < 0.05 vs CCl_4_.

Collagen IV and fibronectin, the two major proteins that compose the basement membrane, are seldom expressed in healthy liver tissues. Immunohistochemical labeling revealed that these two proteins were remarkably elevated in the group with CCl_4_-induced hepatofibrosis and diminished in the group treated with olmesartan (Figures 4(a) and 4(b)).

### 3.4. Olmesartan's Effects on Intrahepatic Angiogenesis in Fibrotic Mice through the AT1R/VEGF Pathway

**Figure 5:**
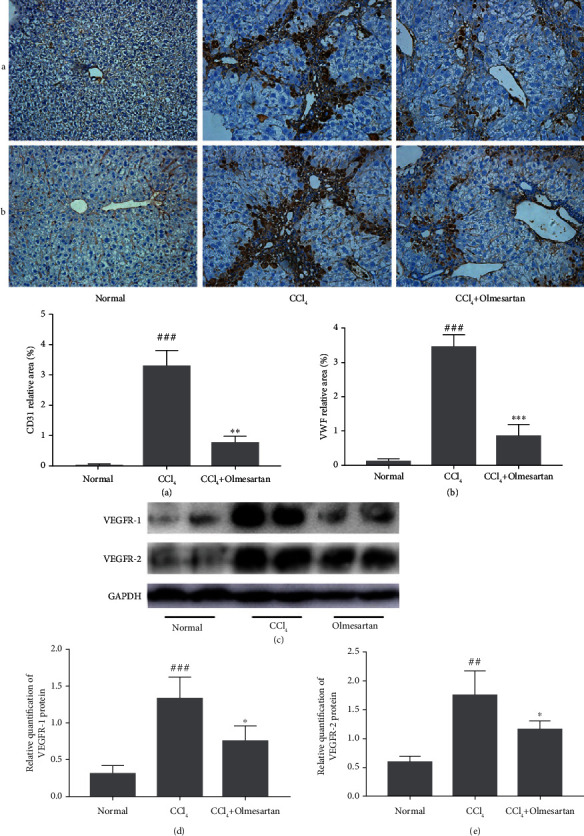
The impact of olmesartan on intrahepatic angiogenesis in mice with fibrotic livers. (a, b) Immunohistochemical staining of CD31 and VWF in liver tissues of mice (magnification ×200). (c–e) Utilizing a western blot technique, the expression levels of VEGFR-1 and VEGFR-2 in liver tissues were determined. ^###^*P* < 0.001 versus normal; ^##^*P* < 0.01 versus normal; ^∗∗∗^*P* < 0.001 versus CCl_4_; ^∗∗^*P* < 0.01 versus CCl_4_; ^∗^*P* < 0.05 versus CCl_4_.

Inhibition of intrahepatic angiogenesis has become a new target of drug therapy for liver fibrosis and portal hypertension. CD31 and VWF, which are common vascular endothelial markers, are seldom seen in the hepatic sinusoids of a healthy liver. Immunohistochemical staining indicated that CD31 and VWF expression was much higher in the CCl_4_ group than that in other groups. However, the expression of these two proteins was reduced after olmesartan treatment, indicating that olmesartan inhibited CCl_4_-induced intrahepatic angiogenesis (Figures 5(a) and 5(b)).

VEGF significantly facilitates endothelial cell proliferation and intrahepatic angiogenesis. VEGFR-1 and VEGFR-2 are two tyrosine kinase receptors with high affinity that are principally responsible for VEGF activity. The western blot results indicated that VEGFR-1 and VEGFR-2 protein expression in CCl_4_-induced fibrotic livers was notably increased, while the protein expression levels of these two receptors in the olmesartan treatment group were downregulated (Figures 5(c)–5(e)).

**Figure 6:**
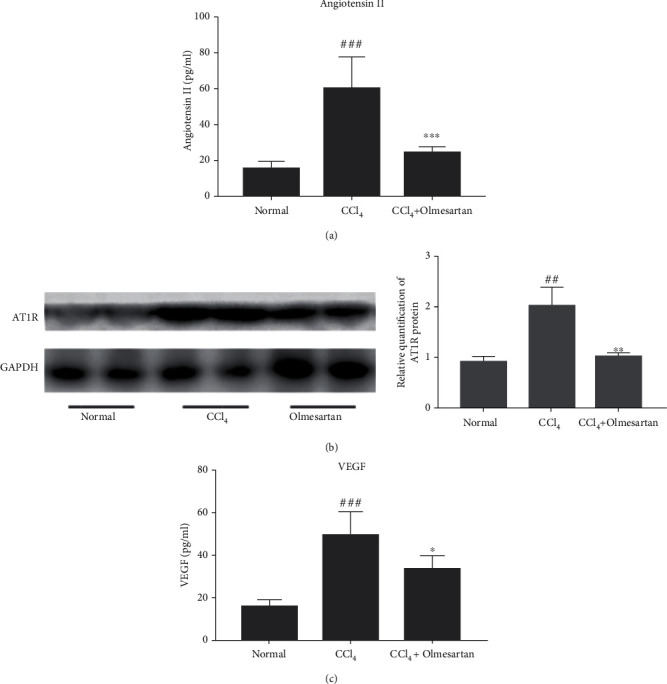
The impact of olmesartan on the AT1R/VEGF pathway in mice with hepatic fibrosis. (a) Serum levels of angiotensin II were measured. ^###^*P* < 0.001 versus normal; ^∗∗∗^*P* < 0.001 versus CCl_4_. (b) Western blot analysis was used to determine the AT1R expression levels in liver tissues. ^##^*P* < 0.01 versus normal; ^∗∗^*P* < 0.01 versus CCl_4_. (c) Serum levels of VEGF were measured. ^###^*P* < 0.001 versus normal; ^∗^*P* < 0.05 versus CCl_4_.

We examined the levels of angiotensin II, AT1R, and VEGF in mice treated with olmesartan. Blood levels of angiotensin II were considerably higher in the CCl_4_-induced fibrosis group compared with the normal group, but clearly lower in the olmesartan group (Figure 6(a)). In addition, we found that CCl_4_ increased hepatic AT1R expression, but olmesartan downregulated angiotensin II-induced AT1R protein expression (Figure 6(b)). In the CCl_4_-induced liver fibrosis group, angiotensin II promoted the production of VEGF, while olmesartan dramatically reduced angiotensin II-induced VEGF production (Figure 6(c)).

## 4. Discussion

Hepatic fibrosis is a pathological condition characterized by excessive ECM production and inflammatory cell infiltration [25]. Cirrhosis, portal hypertension, and even liver cancer may occur from hepatic fibrosis. Sinusoidal capillarization and hepatic angiogenesis have the greatest impact on the development of liver fibrosis and cirrhosis [26]. ARBs have been shown to lessen the severity of liver fibrosis in rats, but the molecular mechanism behind this benefit is not very clear [18, 27]. As a long-lasting ARB, olmesartan has been indicated to be more effective than other ARBs in lowering blood pressure [28, 29]. In our study, the effects of olmesartan on hepatic sinusoidal capillarization and angiogenesis in the liver fibrosis model mice were investigated.

By injecting intraperitoneal CCl_4_ for eight weeks, we were able to successfully generate a mouse model of liver fibrosis. According to pathological staining, olmesartan significantly lowered collagen deposition, inflammation, and fibrosis in mouse liver tissues compared to those in the CCl_4_ group. HSCs have been confirmed to make a crucial impact in the progression of hepatic fibrosis [30]. In addition, *α*-SMA is a common marker for assessing HSC activity and proliferation [31]. When HSCs are activated with CCl_4_, *α*-SMA expression and ECM component deposition increase. Western blot and immunohistochemical staining revealed that olmesartan inhibited *α*-SMA expression and the activation of HSCs. PDGF-BB may enhance the collagen I and *α*-SMA levels, according to our previous studies [5]. PDGF is the most potent proliferation-stimulating factor of HSCs and can promote HSC activation, motility, and migration after binding to its receptor [32]. Therefore, blocking PDGF signaling can alleviate liver fibrosis and angiogenesis [33]. In the present study, the serum PDGF levels in mice in the liver fibrosis group were markedly increased, but olmesartan reduced PDGF expression to attenuate intrahepatic angiogenesis and fibrosis. In addition to regulating inflammatory mediators, IL-10 can promote HSC apoptosis and affect ECM remodeling, thereby inhibiting fibrosis [34–36]. We demonstrated that CCl_4_ treatment lowered serum IL-10 levels in mice, while olmesartan increased IL-10 expression, indicating that olmesartan has potential anti-inflammatory and antifibrotic effects.

Normal LSECs have characteristic fenestrae that are organized into groups of sieve plates, which can maintain liver microcirculation and homeostasis [37]. In chronic liver diseases, LSECs lose the unique fenestrated phenotype and their normal functions; this process is known as capillarization [38]. LSEC capillarization promotes HSC activation and angiogenesis in the progression of hepatic fibrosis [39–41]. In the early stages of liver fibrosis, the crosstalk between LSECs and HSCs promotes angiogenesis and fibrogenesis [33]. In addition, excessive ECM deposition and LSEC capillarization can change liver architecture and increase sinusoidal resistance, ultimately leading to portal hypertension [42]. In our present study, the TEM and SEM results demonstrated that LSEC fenestrae in the CCl_4_ treatment group were decreased in number or disappeared and the basement membrane was formed, which led to capillarization and sinusoid remodeling. In addition, collagen IV and fibronectin, which are basement membrane proteins, were prominently increased in the livers of fibrotic mice, but olmesartan treatment reduced the expression of these proteins and attenuated hepatic sinusoidal capillarization. Moreover, we evaluated the effect of olmesartan on the expression of endothelial cell markers VWF and CD31. Compared with the CCl_4_-treated group, olmesartan downregulated CD31 and VWF protein expression. These findings demonstrate that olmesartan inhibits hepatic angiogenesis and alleviates hepatic sinus pressure and sinusoidal remodeling, which may improve hepatic fibrosis and portal hypertension.

Angiogenesis, the hypoxia-induced formation of new blood vessels from existing ones, is a pivotal stage in the progression of chronic liver disease [43]. VEGF, which is derived from hepatocytes and HSCs, is the most important regulator of angiogenesis and can act on LSECs and HSCs in an autocrine and paracrine manner [44]. VEGF may stimulate endothelial cell proliferation, promote the creation of endothelium tubules, and substantially contribute to the early phases of neovascularization, according to studies [45]. Additionally, LSECs exhibit an increase in VEGFR-1 and VEGFR-2 receptor expression in response to VEGF production [45]. In this study, the expression of VEGF and VEGF receptors was greatly increased in the CCl_4_-induced fibrotic group, while olmesartan decreased both. These results indicate that olmesartan can ameliorate intrahepatic angiogenesis, sinusoidal remodeling, and capillarization by inhibiting VEGF signaling.

As previously established, angiotensin II may promote HSC activation, proliferation, and contraction in vitro. In turn, active HSCs may express RAS components such as AT1R and produce angiotensin II [17]. Studies have reported that angiotensin II may upregulate VEGF to stimulate angiogenesis and that its cellular effects are mediated mainly by AT1R [20, 21]. We investigated the effects of olmesartan on protein levels of VEGF and AT1R in mice with liver fibrosis. AT1R expression was shown to be favorably associated with VEGF expression. Our findings suggested that angiotensin II facilitated HSC activation and upregulated AT1R protein expression, thereby increasing VEGF secretion in HSCs, and that effect was reversed by olmesartan. Therefore, these findings demonstrate that olmesartan, an AT1R antagonist, inhibits angiogenesis in mice with liver fibrosis through the angiotensin II-AT1R-VEGF axis.

## 5. Conclusion

Our findings demonstrate that the AT1R antagonist olmesartan suppresses HSC activation and collagen deposition in liver fibrotic mice and has the effect of anti-angiogenesis and improving hepatic sinusoidal remodeling through the angiotensin II-AT1R-VEGF axis. Olmesartan may be an effective treatment agent for hepatic fibrosis and portal hypertension targeting neovascularization.

## Data Availability

Upon request, the corresponding author may give the data used to support the study's results.
